# Hypoxia-inducible factor-1α activates transforming growth factor-β1/Smad signaling and increases collagen deposition in dermal fibroblasts

**DOI:** 10.18632/oncotarget.23225

**Published:** 2017-12-14

**Authors:** Xu Mingyuan, Pang Qianqian, Xu Shengquan, Ye Chenyi, Lei Rui, Shen Yichen, Xu Jinghong

**Affiliations:** ^1^ Department of Plastic Surgery, The First Affiliated Hospital, School of Medicine, Zhejiang University, Hangzhou, 310003, China; ^2^ Department of Hand Surgery and Microsurgery Center, The First Affiliated Hospital, School of Medicine, Zhejiang University, Hangzhou, 310003, China; ^3^ Department of Orthopedic Surgery, The Second Affiliated Hospital, School of Medicine, Zhejiang University, Hangzhou, 310009, China

**Keywords:** keloid, hypoxia, hypoxia-Inducible factor-1α, transforming growth factor-β1/Smad, collagen

## Abstract

Hypoxia of local tissue occurs during the scar formation; however, the degree of ischemia and hypoxia in the central areas of keloids is more serious than those in normal scars. Hypoxia-induced factor (HIF), is one of the main cellular responses to hypoxia, allowing cells to adapt to low-oxygen conditions. We investigated the correlation among hypoxia, transforming growth factor-β1/Smad signaling and collagen deposition. Hypoxia up-regulated TGF-β1, Smad2/3, p-Smad2/3, Smad4, and total collagen in both normal and keloid fibroblasts via HIF-1α, which was attenuated by HIF-1α inhibition, but TβRII levels were not significantly altered. Silencing Smad4 under hypoxia decreased the mRNA and protein levels of HIF-1α, suggesting up-regulated Smad4 may also plays a role in promoting HIF-1α. Finally, we examined the role of the TGF-β1/Smad pathway in collagen deposition. When TβRII was inhibited by ITD-1 under hypoxic conditions, p-Smad2/3 levels and collagen deposition decreased. When inhibited TβRII by siRNA under normoxia, the levels of p-Smad2/3, Smad4 and collagen deposition also decreased. This result demonstrated that hypoxia promoted TGF-β1/Smad signaling via HIF-1α and that both HIF-1α and the TGF-β1/Smad signaling promotes collagen deposition in hypoxia, which is an important mechanism of keloid formation.

## INTRODUCTION

Keloids are a common type of pathological skin healing that contain excessive fibroblasts with high expression of extracellular matrix (ECM), particularly the excessive synthesis and deposition of collagen and mucopolysaccharides [1]. Keloids exhibit relative hypoxia conditions, with elevated hypoxia-inducible factor-1α (HIF-1α) expression and reduced vascular density compared with normal skin [2]. During the occurrence of keloids, trauma-induced destruction of the skin vascular network, along with the high metabolic state of cells during inflammation and repair, lead to an ischemia-hypoxia state [3, 4]. HIF-1 expression is significantly increased during scar formation and wound healing in humans [5–7]. Animal models have also confirmed that oxygen content in local tissue is significantly decreased during scar formation [3].

HIF-1α is a subunit of the heterodimeric transcription factor hypoxia-inducible factor 1 (HIF-1), which is induced by extremely low oxygen concentrations (0–2%) and functions as a major transcriptional regulator of adaptive responses to hypoxia. Under normal oxygen conditions, prolyl hydroxylase (PHD) can hydroxylate HIF-1α and lead to its rapid degradation, whereas hypoxia blocks this process [8]. Schodel [9] found that HIF-1 up-regulates connective tissue growth factor(CTGF), which is strongly promotes fibroblasts proliferation and ECM synthesis, playing a key role in keloid formation. Moreover, Deng [10] reported that Transforming growth factor-β (TGF-β) levels are up-regulated in cancer cells during hypoxia.

TGF-β is a multifunctional cytokine, which comprises three different isoforms (TGF-β1, TGF-β2, TGF-β3) in mammals, that activates the membrane receptor serine/threonine kinase complex composed of type II (TβRII) and type I (TβRI) receptors. After the binding of TGF-β, TβRII phosphorylates and activates TβRI, leading to the activation of the TGF-β/Smad signaling pathway. The TGF-β/Smad pathway plays a vital role in cell growth, differentiation, apoptosis, and proliferation [11–13]. TGF-β1 is an important member of TGF-β family that has been found to be up-regulated in keloid tissue and is reported to stimulate collagen formation and ECM synthesis and to decrease extracellular matrix degradation [14, 15] and might be involved in the formation of keloids [16]. Hypoxia has been reported to promote TGF-β1 in gastric cancer [10], and TGF-β expression is enhanced in scar tissue fibroblasts [17–19]. TGF-β also has been reported to cooperate with CTGF to induce sustained fibrosis [20].

Fibroblasts can synthesize, deposit and remodel ECM and play a vital role in wound healing [20]. In the present study, we study the response of human dermal fibroblasts to hypoxia, and focused on the effect of TGF-β1/Smad signaling and collagen deposition during hypoxia and the role of HIF-1α and the TGF-β1/Smad pathway in collagen deposition. We hypothesized that hypoxia promotes the TGF-β1/Smad signaling pathway via HIF-1α. And hypoxia increased collagen deposition via HIF-1α and TGF-β1/Smad signaling pathway.

## RESULTS

### Hypoxia promotes TGF-β1/Smad signaling

**Figure 1:**
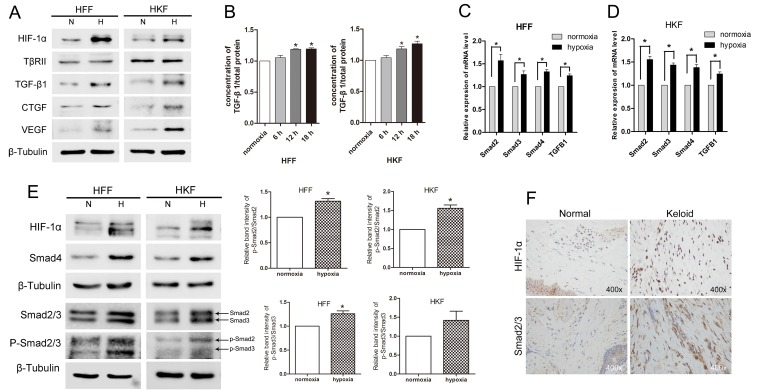
Hypoxia promoted TGF-β/Smad signaling in HFFs and HKFs (**A**) HIF-1α, TGF-β1, VEGF and CTGF protein levels were up-regulated by 24 h of hypoxia (1% O_2_) exposure, but TβRII expression did not obviously differ between the normoxia and hypoxia groups. (**B**) ELISA was used to detect secreted TGF-β1 in serum-free medium after 6 h, 12 h and 18 h of exposure to normoxia (21% O_2_) or hypoxia (1% O_2_). (**C**, **D**) Quantitative reverse transcriptase-PCR (qRT-PCR) analyses of Smad2, Smad3, Smad4 and TGF-β1 mRNA levels in HFFs and HKFs after 24 h of hypoxia or normoxia exposure. Bars show the means±SE of three independent experiments (*n* = 3); ^*^represents *P* < 0.05. (**E**) Western blotting shows the protein levels of HIF-1α, Smad2/3, p-Smad2/3 and Smad4 after 24 h of hypoxia or normoxia exposure. The histogram shows the protein band intensity ratio of p-Smad2 to Smad2 and p-Smad3 to Smad3. (**F**) The expression of HIF-1α and Smad2/3 was tested using immunohistochemistry analysis.

**Figure 2:**
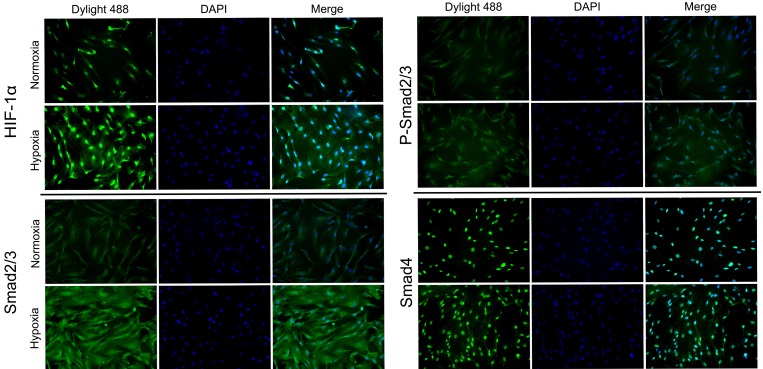
HIF-1α, Smad2/3, p-Smad2/3 and Smad4 was enhanced following treatment with 1% hypoxia The protein expression and intracellular localization of HIF-1α, Smad2/3, p-Smad2/3 and Smad4 were detected by immunofluorescence staining in HFFs under normoxia or 24 h of hypoxia (1% O_2_). HIF-1α and Smad4 localized mainly in the nucleus and Smad2/3 mainly in cytoplasm.

To analyze the effect of hypoxia in fibroblasts, we detected the expression of mRNA and proteins in both HFFs and HKFs under normoxia (21% O_2_) and hypoxia (1% O_2_). Western blotting (Figure 1A) revealed that HIF-1α, CTGF and vascular endothelial growth factor (VEGF) were obviously up-regulated after 24 h under hypoxia. Then we investigated whether acute hypoxia promoted the TGF-β1/Smad pathway. The levels of both intracellular and secreted TGF-β1 were significantly higher in HFFs and HKFs from the hypoxia group compared with the normoxia group; however, TGF-β receptor type II (TβRII) showed no obviously difference (Figure 1A–1B). As shown in Figure 1C–1E, Smad2/3 and Smad4 mRNA and protein levels were up-regulated by 24 h of hypoxia. It has been demonstrated that TGF-β1 binds to the TβRII to activate the TGF-β receptor type I (TβRI) kinase, resulting in the phosphorylation of Smad2 and Smad3, which subsequently form oligomeric complexes with Smad4 and translocate into the nucleus [21, 22]. In this study, we found the protein level of the phosphorylation of Smad2/3 (p-Smad2/3) and ratio of p-Smad2 to Smad2 and p-Smad3 to Smad3 were also up-regulated in hypoxia condition, indicating the activation of Smad2/3. Immunohistochemistry staining (Figure 1F) showed higher protein levels of HIF-1α and Smad2/3 in keloid dermis than those in normal dermis. Immunofluorescence staining (Figure 2) revealed increased of HIF-1α protein and nuclear localization under hypoxia, and the amounts of Smad2/3, p-Smad2/3 and Smad4 protein were up-regulated.

### siHIF-1α inhibits TGF-β1/Smad signaling in hypoxia

**Figure 3:**
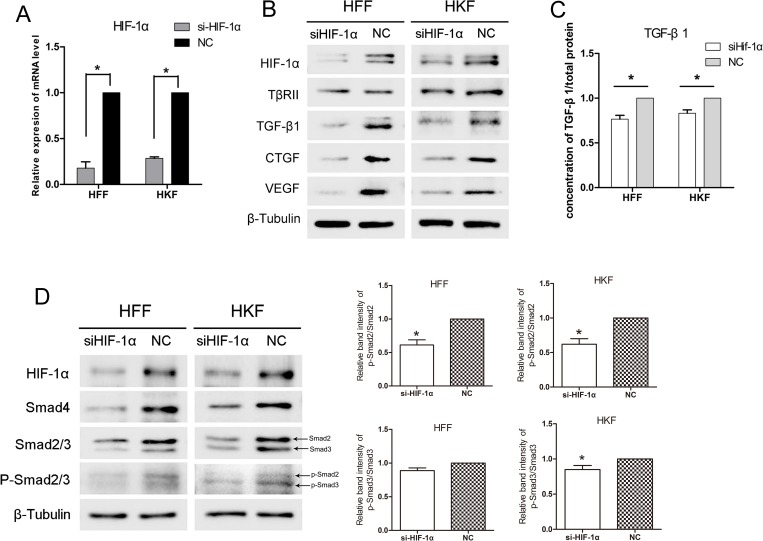
siHIF-1α inhibited TGF-β/Smad signaling in HFFs and HKFs (**A**) qRT-PCR showing clear knockdown of HIF-1α by siHIF-1α transfection for 48 h. (**B**, **D**) 48 h after transfection, HFFs and HKFs were transferred to a 1% O_2_ hypoxia incubator for 24 h, and siHIF-1α inhibited HIF-1α, TGF-β1, Smad4, Smad2/3, p-Smad2/3, CTGF and VEGF levels. The histogram shows the protein band intensity ratio of p-Smad2 to Smad2 and p-Smad3 to Smad3. (**C**) Before transferring cells to the hypoxia incubator, we replaced the culture media with serum-free media, and both the siHIF-1α and NC groups were treated with 1% O_2_ for 12 h. The level of secreted TGF-β1 in the serum-free media was then measured.

Given that hypoxia up-regulates HIF-1α and the TGF-β/Smad pathway, we then investigated whether the influence on TGF-β pathway was caused by HIF-1α. To this end, we silenced HIF-1α using small interfering RNA (siRNA) and exposed HFFs and HKFs to hypoxia conditions for 24 h. Figure 3A, 3B shows the clear knockdown of HIF-1α at both the mRNA and protein level in HFFs and HKFs. The proteins in TGF-β/Smad pathway were also inhibited by siHIF-1α. The intracellular and secreted levels of TGF-β1, CTGF, and VEGF decreased in the siHIF-1α group, whereas TβRII was unaltered (Figure 3B–3C).

siHif-1α transfection also reduced the positive effect of hypoxia on Smad2/3, p-Smad2/3 and Smad4 (Figure 3D), suggesting that hypoxia up-regulates the TGF-β/Smad pathway via HIF-1α.

### siSmad4 inhibits HIF-1α in hypoxia

**Figure 4:**
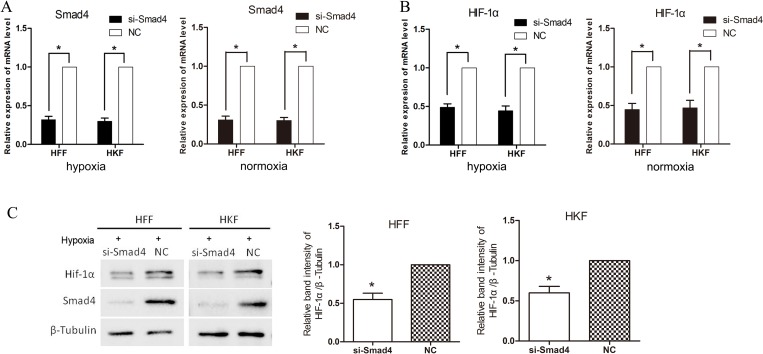
siSmad4 inhibits HIF-1α in hypoxia (**A**) qRT-PCR showing clear knockdown of Smad4 by siSmad4 transfection for 48 h. (**B**) 24 h after transfection, HFFs and HKFs were treated with 1% hypoxia or normoxia for 24 h. 48 h after transfection, the expression of mRNA level of HIF-1α was down-regulated in the group silencing Smad4. (**C**) After 72 h transfected by si-Smad4 and 24 h hypoxia treatment, the protein level of HIF-1α was down-regulated in the si-Smad4 group compared with the negative control group. The histogram shows the protein band intensity ratio of HIF-1α to β-Tubulin.

To investigate the effect of transcription factor, Smad2/3/4 complex, we transfected siRNA into HFFs and HKFs to silence Smad4, then we moved the cells into 1% O_2_ hypoxia incubator for 24 h. Figure 4 shows that on both mRNA and protein levels, HIF-1α was inhibited by siSmad4.

### Hypoxia promotes collagen deposition via HIF-1α and the TGF-β1/Smad pathway

**Figure 5:**
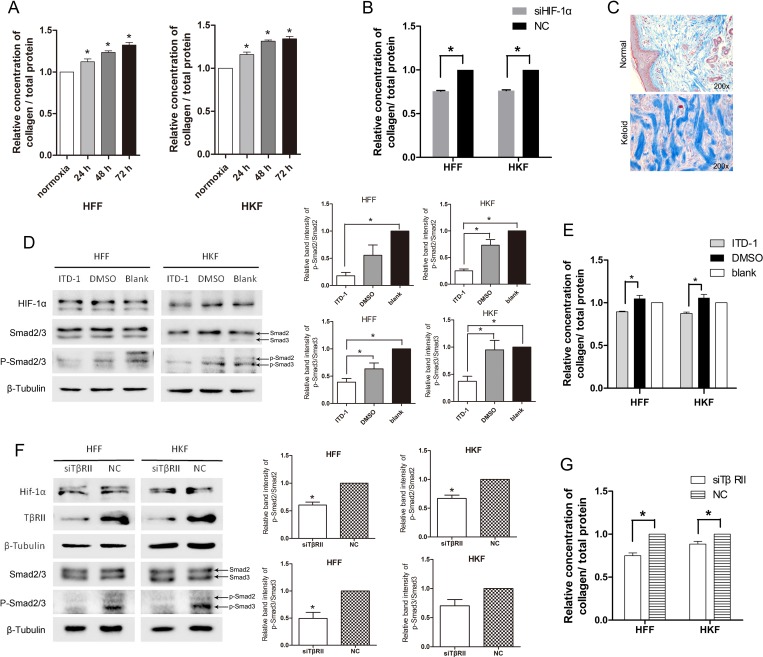
Total collagen deposition was promoted by acute hypoxia via HIF-1α and the TGF-β signaling pathway (**A**) The ratio of deposited collagen to the total protein concentration was elevated after 24 h, 48 h, 72 h of hypoxia in both HFFs and HKFs. (**B**) At 48 h after transfection, HFFs and HKFs were transferred to a 1% O_2_ hypoxia incubator for 24 h. Collagen deposition was clearly inhibited in the siHIF-1α group compared with the NC group. (**C**) Masson staining for collagen fiber (blue) of normal and keloid tissues. (**D**) Treatment with 5 μM ITD-1(dissolved in DMSO), DMSO, and blank, respectively, for 24 h under 1% O_2_ condition. ITD-1 inhibited the phosphorylation of Smad2/3 by inhibiting TβRII. Meanwhile, HIF-1α and Smad2/3 levels remained stable in all three groups. The histogram shows the protein band intensity ratio of p-Smad2 to Smad2 and p-Smad3 to Smad3. (**E**) Collagen deposition was reduced in the ITD-1-treated HFFs and HKFs. (**F**) siTβRII reduced the protein levels of TβRII, p-Smad2/3 at 72 h after transfection in normoxia. HIF-1α protein levels remained stable in the two groups. The histogram shows the protein band intensity ratio of p-Smad2 to Smad2 and p-Smad3 to Smad3. (**G**) Collagen deposition was clearly inhibited in the siTβRII group compared with the NC group.

Excessive formation of collagen fibrils is one of the most significant features for keloid. Figure 5C shows the thick and disorganized collagen fibrils of keloid. TGF-β1 has been reported to induce matrix production through Smad3-dependent mechanisms [23]; therefore, we investigated whether acute hypoxia promotes collagen deposition via HIF-1α and the TGF-β1/Smad pathway. We detected collagen deposition using the Sircol Soluble Collagen Assay. Figure 5A shows that total collagen deposition increased as the time of hypoxia extended for both HFFs and HKFs. Reduced collagen deposition was also detected in the siHIF-1α group after 72 h of transfection (Figure 5B). These results suggested that hypoxia promotes collagen deposition via Hif-1α.

We then studied the role of the TGF-β1/Smad pathway in collagen deposition under hypoxia. ITD-1 (MedChem Express, HY-12704, USA) is a highly selective inhibitor of TβRII [24] that induces TβRII degradation. To investigate whether collagen deposition was regulated by the TGF-β pathway, we inhibited TβRII using ITD-1 under hypoxia. Cells were treated by adding 5 μM ITD-1 to the culture media and cultured under hypoxia for 24 h. The effect of inhibition was detected via the p-Smad2/3, which are downstream targets of the TGF-β receptor. As shown in Figure 5D, the p-Smad2/3 and the ratio of p-Smad2 to Smad2, p-Smad3 to Smad3 clearly reduced in the ITD-1 group, while HIF-1α levels were stable, indicating that activation of Smad2/3 was inhibited by ITD-1. We then examined collagen deposition in the ITD-1, DMSO (i.e., solvent control) group and the blank group. We found that collagen deposition was reduced following inhibition with ITD-1 (Figure 5E). In Figure 5F, 5G, we used siRNA to inhibit TβRII in normoxia and found siTβRII reduced the levels p-Smad2/3 and collagen deposition, with the same trend as the ITD-1 treatment group. It showed that the inhibition of TβRII reduced protein levels of p-Smad2/3, the ratio of p-Smad2 to Smad2, p-Smad3 to Smad3 and collagen deposition.

These results suggest that hypoxia promotes collagen deposition via HIF-1α and the TGF-β1/Smad pathway.

## DISCUSSION

The essence of keloids is the enhanced proliferation and activity of fibroblasts, resulting in excessive increases in the synthesis of ECM, of which collagen is the major component [24]. Keloids exhibit relative hypoxia conditions, as its microvasculature is obstructed due to microvascular endothelial cell proliferation [25, 26]. HIF-1α is significantly increased in scar formation and wound healing in humans [5–7]. Similarly, animal models have also confirmed that oxygen content in local tissues is significantly decreased during scar formation [3]. As a transcriptional activator, HIF-1α can induce the expression of hundreds of genes target genes, which promote angiogenesis and enhance hypoxia tolerance [27]. Unlike other cell lines, both HFFs and HKFs show strong tolerance to hypoxia. HepG2 and PC12 cells, display increased HIF-1 protein levels after 3–6 h of hypoxia exposure [28, 29] and begin to detach and die after 12 h of anoxia exposure. However, in our investigation of hypoxia time, we found that HFFs and HKFs remained firmly adhered without obvious shedding and cell death after 120 h of hypoxia exposure. Furthermore, HIF-1 protein levels started increasing after 6 h of hypoxia exposure, and 12 h of exposure lead to a steady increase [30]. This high tolerance at very low oxygen concentrations is a further research direction for answering why keloids continue to grow in long-term relative hypoxia and nutrient-deficient environments.

TGF-β1 has been shown to play an important role in scar formation and is increased in a variety of cells cultured in hypoxia and in hypoxic ischemic tissue [10, 31]. TGF-β1 promotes scar growth mainly via TβRII and the downstream activation of Smads phosphorylation and nuclear translocation to regulate the expression of a variety genes, specifically regulating fibroblast proliferation and protein synthesis. Hypoxia has been reported to activate latent TGF-β1 in a HIF-dependent manner in hepatocytes [32]. And Shi [33] found hepatic stellate cells LX-2 hypoxia increase the matrix metalloproteinase 2, which process TGF-β to its active form. Meanwhile, the increased level of TGF-β protein was detected in placental fibroblasts [34], primary human lung fibroblasts [35] and smooth muscle cells [36].

In our research, in addition to the increased protein level of TGF-β1, we found the protein level of Smad2/3, p-Smad2/3, Smad4 also increased under hypoxic condition. However, the levels of TβRII were not affected. Hence, it is the increased TGF-β1 that leads to the activation of downstream pathways. Furthermore, ratio of p-Smad2 to Smad2 and p-Smad3 to Smad3 increased in hypoxia, which reveals the activation of TGF-β1/Smad signaling pathway. HIF-1 and Smad3 have been reported to directly interact and synergistically regulate VEGF gene expression [37] and Sp1 [38]. ChIP-Seq revealed 614 sites that are co- occupied by HIF-1α and Smad3, and hypoxia promotes Smad3 binding via HIF-1α [39].

Smad4 binds phosphorylated Smad2/3 to form the Smad2/3/4 complex, which plays an important role in regulating gene transcription. Immunofluorescence showed the Smad4 mainly localized to the nucleus and was also promoted by hypoxia. This complex has been reported to increase the expression of a variety of cytokines, including connective tissue growth factor (CTGF) [18, 40]. In our study, the mRNA and protein levels of HIF-1α decreased when silencing Smad4. Whether there is a binding site for Smad2/3/4 complex on HIF-1α promoter and whether there is a positive feedback loop for HIF-1α and Smad4 need further studies. On the other hand, TGF-β1 was reported to promote the transcription of HIF-1 [19], and HIF-1 directly increases the expression and secretion of CTGF [9]. In the present study, CTGF levels increased under hypoxia, and the deposition of collagen was enhanced, which was consistent with the role of CTGF in promoting collagen secretion in fibroblasts.

The response of collagen deposition to hypoxia varies with cells. hypoxia significantly reduced collagen I secretion in human corneal fibroblasts from keratoconus patients [41]. However, in dermal fibroblast collagen increased under hypoxic condition [42, 43]. And Zhao [43] found it is associated with the activation of Smad3. As for animal experiments, six-week hypoxic exposure (10% O_2_) significantly increased collagen I and III in control nontransgenic mouse lung, but it had no significant effects on mouse models that expresses an inducible dominant negative mutation of TβRII [44]. In our study, using ITD-1 and si-TβRII to block TβRII led to the decreased the ratio of p-Smad2/3 to Smad2/3 and collagen deposition without affecting total Smad2/3 levels. So in hypoxia, the up-regulated TGF-β1 binds the TβRII and promotes the collagen depositon.

The above phenomena indicate that under hypoxic stress, the transcription and expression of proteins that promote cell growth and proliferation, including CTGF, TGF-β1, Smad2, Smad3 and Smad4, were enhanced, thereby promoting cell growth, proliferation and collagen deposition. At the same time, we found that hypoxia up-regulated p-Smad2/3 and Smad4 via HIF-1α, and blocking TβRII reduced the p-Smad2/3 and collagen deposition, suggesting that hypoxia promotes TGF-β1/Smad signaling and then the up-regulated TGF-β1 and p-Smad2/3 raises collagen deposition. And the up-regulated Smad4 may promotes HIF-1α.

In keloid tissue, the elevated level of HIF-1α protein caused by more serious hypoxia conditions may activate the TGF-β1/Smad pathway, whereupon both HIF-1α and the TGF-β1/Smad pathway act to promote collagen deposition during keloid formation.

## MATERIALS AND METHODS

### Cell culture

Human foreskin fibroblasts(HFFs) cells were purchased from the American Type Culture Collection (ATCC^®^ SCRC1041™). Primary human keloid fibroblasts (HKFs) were isolated from discarded keloid tissues from patients (yellow race, female, 25-year-old, keloid of earlobe) who were undergoing surgery. Both cell types were cultured in DMEM(GNM12800-S, Hangzhou, China) containing 100μg/ml streptomycin and 100 U/ml penicillin and supplemented with 12% fetal bovine serum(Gibco,10099-141) at 37°C, under a 5% CO_2_ atmosphere. Hypoxia was induced by culturing fibroblasts in a hypoxia incubator set at 1% O_2_ in a 5% CO_2_ humidified environment at 37°C.

### Human tissue specimens

Keloid specimens were harvested from typical keloid patients (yellow race, female, 25-year-old, keloid of earlobe) at the time of surgical excision, while normal skin specimens were obtained from patients (yellow race, female, 37-year-old, skin of forehead) who underwent surgical procedures for cosmetic reasons and displayed no keloid, hypertrophic scars or current infections. All procedures in this study were approved by the Ethics Committee of the First Affiliated Hospital, College of Medicine, Zhejiang University, and all patients provided written informed consent. All experiments were performed in accordance with relevant guidelines and regulations.

### Immunohistochemistry

Biopsy specimens were fixed immediately after resection, placed in 10% formalin saline for 24 hours and dehydrated by standard histological procedures. For immunohistochemistry staining, rabbit monoclonal antibodies directed against HIF-1α (ab51608, Abcam, USA; dilution 1:200) and Smad2/3 (#8685, CST, USA; dilution 1:100) were used and horseradish peroxidase-conjugated goat anti-rabbit/mouse IgG (DAKO, K5007, Denmark) acted as the secondary antibody. Staining was achieved using a DAB Stain kit (DAKO, K5007, Denmark).

### Construction of small interfering RNA (siRNA) and celltransfection

The siRNA targeting HIF-1α, Smad4 and TβRII messenger RNA (mRNA) and a scrambled siRNA used as a negative control (NC) were synthesized by OriGene Technologies(USA). Three effective siRNAs for one gene were mixed to reduce off-target effects. Cells were transfected using Lipofectamine2000 Reagent (Invitrogen, USA) according to the manufacturer’s instructions. The transfected cells (HFF-1 and HKFs) were cultured for 48 h for qRT-PCR and 72 h for Western blotting. The efficiency of gene knockdown was detected by quantitative real-time polymerase chain reaction (qRT-PCR).

### RNA extraction and real-time qRT-PCR

RNAiso plus (Takara, Dalian, China) was used to extract total RNA from the cells. cDNA was synthesized from 600 ng of total RNA using the PrimeScript RT reagent Kit (Takara, Dalian, China). mRNA expression was measured by real-time PCR on a CFX96™ Real-Time System (Bio-Rad) using the SYBR-Green method (Takara, Dalian, China) according to the manufacturer’s instructions. The primer sequences are listed in Table 1. The expression levels were normalized to β-tubulin and measured using the comparative threshold cycle (ΔΔCt) method.

**Table 1:** Oligonucleotide primer sets for real-time PCR

Name	Sequence(5′¬3′)
β-tubulin-F	AAGATCCGAGAAGAATACCCTGA
β-tubulin-R	CTACCAACTGATGGACGGAGA
TGF-β1-F	CTAATGGTGGAAACCCACAACG
TGF-β1-R	TATCGCCAGGAATTGTTGCTG
Smad2-F	CGTCCATCTTGCCATTCACG
Smad2-R	CTCAAGCTCATCTAATCGTCGTCCTG
Smad3-F	TGGACGCAGGTTCTCCAAAC
Smad3-R	CCGGCTCGCAGTAGGTAAC
Smad4-F	CTCATGTGATCTATGCCCGTC
Smad4-R	AGGTGATACAACTCGTTCGTAGT

^*^F, forward primer; R, reverse primer.

### Protein extraction and western blotting

Total protein was extracted from confluent cell cultures using RIPA Lysis Buffer, Strong (P0013B Beyotime) supplemented with protease inhibitor cocktail (G2006, Servicebio, China). Equal amounts of protein were separated via 8% SDS-PAGE and then transferred to PVDF membranes. Membranes were blocked with 5% BSA at room temperature. The following antibodies were used: anti-HIF-1-alpha antibody (ab51608, Abcam, USA; dilution 1:1000);anti-beta tubulin monoclonal antibody (E021040, EarthOx, USA, dilution 1:4000); anti-TGF beta receptor II antibody(ab186838, Abcam, USA; dilution 1:1000); Smad2/3 (D7G7) XP Rabbit mAb(#8685, CST, USA; dilution 1:2000); p-Smad2/3 antibody (#8828, CST, USA; dilution 1:1000); Smad4 antibody (#9515, CST, USA; dilution 1:1000); Smad7 (ab55493, Abcam, USA; dilution 1:1000); anti-TGF beta 1 antibody (ab155264, Abcam, USA, dilution 1:1000); HRP affiniPure goat anti-rabbit IgG(H+L) (E030120, EarthOx, USA; dilution 1:5000), and rabbit anti-mouse IgG H&L (HRP) (ab6728, Abcam, USA; dilution 1:5000). Secondary antibody was applied for 1 h at room temperature.The membranes were developed using an ECL Prime Western blotting detection reagent (GE Healthcare). Detection of immunoreactive proteins was performed using the SuperLumia ECL Plus HRP Substrate Kit(Abbkine, K22030) for HIF1A and p-Smad2/3 CTGF, VEGF, TGF-β1,and the SuperSignal™ West Pico Chemiluminescent Substrate(Thermo Scientific, #1856135) for all other proteins (Amersham Pharmacia Biotech Inc.). β-tubulin was used as a loading control.

### Collagen detection

Collagen deposition was detected using the Sircol Soluble Collagen Assay (S100, Biocolor, UK) according to the manufacturer’s instructions. Collagen deposits were extracted according to the same procedure describe above for total proteins.

### ELISA for TGFβ1

After being placed in serum-free medium, cells were cultured in a hypoxia or normoxia incubator for 12 h. The culture medium was then collected for the detection of secreted TGF-β1 using an Enzyme-linked Immunosorbent Assay Kit for TGF-β1 (Cloud-Clone Corp, China).

### Immunofluorescence staining

After treatment with 1%O_2_ or 21% O_2_ for 24 h, HFFs were cultured on sterile glass slides in 6 cm culture dishes. After three washes with PBS, the cells were fixed with 4% formaldehyde for 15 min and permeabilized with 0.1% TritonX-100. The cells fixed on glass slides were blocked with 5% BSA and then incubated overnight with primary antibodies against HIF-1α, Smad2/3, Smad4, TβRII (as above for Western blotting, 1:200 dilution) at 4°C overnight. After three washes with PBS, the glass slides were incubated with Dylight 488 goat anti-rabbit IgG (E032220-01, EarthOx) for 1 h at room temperature. Nuclei were then counterstained with 4′,6-diamidino-2-phenylindole (DAPI). A fluorescence microscope was used to acquire cells images and observe protein localization.

### Statistical analysis

Statistical analyses were performed using Student’s *t*-test to identify differences between groups. *P* < 0.05 was considered statistically significant. All experiments have been repeated for three times, if not mentioned particularly in figure legends.

## Abbreviations

HIF-1α: hypoxia-inducible factor-1α
TGF-β: transforming growth factor-β
TβRI: transforming growth factor-β receptor type I
TβRII: transforming growth factor-β receptor type II
ECM: extracellular matrix
PHD: prolyl hydroxylase
CTGF: connective tissue growth factor
VEGF: vascular endothelial growth factor
siRNA: small interfering RNA
HFFs: human foreskin fibroblasts
HKFs: human keloid fibroblasts
